# Policy development, implementation and evaluation by the AIDS control program in Uganda: a review of the processes

**DOI:** 10.1186/1478-4505-11-7

**Published:** 2013-02-23

**Authors:** Benson T Tumwesigye, Damalie Nakanjako, Rhoda Wanyenze, Zainab Akol, Nelson Sewankambo

**Affiliations:** 1 STD/AIDS Control Program, Ministry of Health Uganda, P.O. Box 7272, Kampala, Uganda; 2 School of Public Health, Makerere University College of Health Sciences, P.O. Box 7072, Kampala, Uganda; 3 School of Medicine, Makerere University College of Health Sciences, P.O. Box 7072, Kampala, Uganda

**Keywords:** Health policy, Policy development, Policy processes, Policy revision

## Abstract

**Background:**

The AIDS Control Program (ACP) in Uganda has spearheaded the national health sector HIV response for the last three decades. ACP has developed, revised and implemented various HIV prevention, care and treatment policies in order to keep interventions relevant to the changing dynamics of the HIV epidemic. However, the ACP team and partners remain concerned about the lengthy policy development processes. This study documented the policy development and revision processes to identify strengths and weaknesses in order to inform adjustments as Uganda embraces the move to ‘zero’ HIV infections.

**Methods:**

Data was collected through a review of the relevant policy documents and key informant interviews with the five program officers involved in the recently developed Safe Male Circumcision (SMC) policy and the recently revised HIV Counseling and Testing (HCT) policy. Qualitative data was analyzed manually using pre-determined themes.

**Results:**

Development and revision of the SMC and HCT policies followed similar processes that included a series of meetings between senior management and a selected technical working group. However, the gaps included: i) inadequate awareness of the existence of national policy development and management guidelines; ii) limited engagement of the policy analysis unit in the policy development/revision processes; iii) inadequate tracking and evaluation of the policies before revision or development of new related policies; iv) lack of specific protocols/standard operating procedures (SOPs); and, v) limited indigenous funding for the entire policy development processes which contributed to non-adherence to the anticipated timelines.

**Conclusions:**

Policy development and revision of the SMC and HCT policies followed similar processes. Gaps identified included lack of protocols/SOPs for the processes and limited indigenous funding to support adherence to anticipated timelines. We recommend active involvement of the policy analysis unit in all policy processes. Specific protocols/SOPs for development, analysis, revision, implementation, monitoring, evaluation and impact assessment processes should be developed prior to commencement of the activities.

## Introduction

Over the last three decades, the response to the HIV/AIDS epidemic in Uganda has led to a reduction in HIV prevalence from 18% in the early 1980s to 7.3% in 2011 [1]. Significant achievements have been realized in some indicators; for example knowledge of HIV sero-status among adult women increased from 13% in 2004 to 66% in 2011, and from 11% to 45% among adult men [1]. The Uganda HIV response is attributable to both national and international commitment to control the global HIV epidemic in Africa. As part of the national health sector HIV response in Uganda, the AIDS Control Program (ACP) has developed and revised several HIV/AIDS prevention, care and treatment policies to improve the lives of people living with HIV/AIDS. However, the ACP team and partners remain concerned about the fact that the policy development/revision processes often take longer than the anticipated period. Subsequently, these delays affect timely implementation of critical evidence-based interventions and may partly explain the current stagnation of HIV prevalence [1]. With the move to ‘zero’ HIV infections, Uganda needs to document and review previous successes and challenges faced in HIV policy development and review processes in order to inform the formulation of strategies to strengthen policy development/revision systems in ACP and the entire Ministry of Health (MoH).

Policies arise from systematic processes to build support for public health action through integration of available evidence, community preferences, political realities and resource availability [2]. Whereas a lot has been documented about the generic methods of research utilization in policy making [3-8], there is limited evidence on the actual protocols followed during the policy development/review processes in resource-limited settings. In this study, we documented the processes that were taken by the ACP technical program officers in the policy development and revision processes with specific attention to the recently revised HIV Counseling and Testing (HCT) and the new Safe Male Circumcision (SMC) policies. Dissemination of these findings locally and internationally is important to inform HIV policy development processes in Uganda and other sub-Saharan countries grappling with developing strategic policies in their national and global HIV response.

## Methods

The project was implemented from the ACP of the MoH which has the mandate to develop/revise HIV/AIDS prevention, care and treatment policies in Uganda. Data on the health policy development process was collected through a retrospective review of MoH ACP policy documents and reports on the recently developed SMC policy [9] and the 2011 HCT policy, selected as case studies to allow easy recall of the processes involved. In addition, key informant interviews were conducted with five ACP technical/program officers who gave verbal informed consent to participate in this evaluation. These program officers were purposively selected given that they were involved in the policy development/revision processes for the new SMC and the 2011 HCT policies. The key informant interview guide covered pre-determined themes that included presence and utilization of guidelines, activities performed, timelines, challenges and proposed solutions to improve policy development/review systems. Qualitative data, from open-ended questions, was analyzed manually according to the predetermined themes that included awareness and utilization of national guidelines, duration of policy development/revision processes, challenges and suggestions for improvement.

## Results

### Policy development and review processes

Most of the ACP program officers interviewed were not aware of the existence of the national policy development and management guide [10]. One member was aware that these guidelines existed but mentioned that they were not always consulted (Table 1). Similarly, one program officer mentioned: ‘*The policy analysis unit was hardly consulted*’, yet they were supposed to be consulted on all MoH policy matters including policy development processes. However, the HCT and SMC followed similar processes that included consultative meetings between MoH senior management and a selected technical working group (TWG), followed by approval and launch by senior management (Table 2). The program officers mentioned that there was no documented comprehensive evaluation of previous policies before revision ensued. Furthermore, the program officers mentioned that there was no documented monitoring and evaluation plan for the policy processes in the ACP: ‘*Most of the current policy evaluation processes are informal and are mainly driven by specific program goals’*. It was however noted that most policies had a provision for consultation of various stakeholders as required.

**Table 1:** Prevalent policy development and implementation processes at the national Ministry of Health and AIDS control program in Uganda

**Policy development and implementation process as reported by the program officers**	
**Presence of guidelines**	
	Written guidelines of policy the development were present
	Adherence to documented processes was not documented
	Monitoring and evaluation of the policies was informal and driven by other stakeholders
**Duration of policy development and Implementation process (in years)**	
	All officers mentioned that process took 1–3 years; longer than the anticipated 6–8 months
	**Reasons cited for the delayed period**
	-Inadequate capacity in terms of knowledge & skills
	-Unclear guidelines
	-Inadequate specific funding for the activities
	-Busy technical working group members
**Importance of guidelines**	
	All respondents indicated that it was timely and important to have a policy process framework or protocol tailored to the ACP Uganda.
	One respondent indicated that this would also apply for the other departments within the MoH
**Monitoring and evaluation**	
	Four out of five reported involvement of a consultant in the policy-making process

**Table 2:** Policy development and review processes for the HIV Counseling and Testing (HCT) and Safe Male Circumcision (SMC) policies at the Ministry of Health, Uganda

	**Revision of HCT policy**	**Medical male circumcision new policy development**
**Duration of policy development and implementation process**	2 years (planned duration to completion was 8 months)	1 year (planned duration was 6 months)
**Presence of guidelines**	No specific guidelines followed	No specific guidelines followed
**Technical working team (15)**	Leader: DG, MoH; technical staff, implementing partners, development agencies, academicians, and CSO	SMC National Task Force (NTF) for HIV prevention included senior MoH technical staff members, development partners, implementing partners and CSO
**Technical team selection**	Based on previous experience in policy development engagement, experience in HCT implementation	Appointed by the Director General MoH Team included experienced persons in HIV response
**Technical working group meeting**	Several held to produce draft 0	Consultant hired right from the beginning to produce draft 0
**Consultative meetings**	Held to develop drafts 1 and 2	Held to produce draft 2
		-Stakeholders meeting produced draft 3
**Peer review by technical team**	Develop draft 3	NTF reviewed draft 0 to produce draft 3
Senior management committee review	Develop draft 4	NTF forwarded final report tosenior management that developed draft 4
Director general of Health Services	Endorsed draft 4	Endorsed draft 4
Launching	Launched by MoH before public use	
**Challenges**	Limited indigenous funding for policy development activities	Limited policy development activities
	Busy TWG members	

DG: Director General of Health Services; TWG: Technical Working Group; CSO: Civil Society Organizations.

### Duration of policy development processes

All program officers indicated that most of the policies took 1 to 3 years which is longer than the anticipated 6 to 8 months, based on the terms of reference (TOR). Delays were associated with lack of clear guidelines and inadequate funding of the processes. The program officers highlighted the challenge of limited funds to support enough policy-related activities such as consultative meetings, consultants’ fees, technical working group meetings, massive printing of policy copies and policy dissemination, as well as monitoring and evaluation of policy implementation processes. They further indicated that these delays were likely to affect the timely and effective implementation of the policies. All officers reported absence of protocols/standard operating procedures (SOPs) and documentation of actual processes taken for each policy. Four out of five respondents indicated that they engaged a consultant for technical advice in the policy making process (Table 1).

In addition, we identified competing responsibilities among the technical working group (TWG) members as a key factor in the duration of the policy development/revision processes. It was indicated that the persons appointed on the TWG were full-time employees of the MoH, academic institutions, implementing partners and development agencies. This, according to the respondents, affected the time committed to the policy development process since the TWG often did not meet on the scheduled time due to other competing responsibilities.

### SMC policy development and HCT policy revision

Overall, the main steps taken in the development and revision of the SMC and HCT, respectively, were similar, including series of meetings between senior management and a selected TWG. Four drafts were produced for each of the policies although the detailed processes differed in terms of the duration of the process, selection of the committee and the number of meetings held. Revision of the HCT policy took two years although the anticipated duration had been eight months as per the TOR. On the other hand, development of SMC took one year despite the anticipated six months as per the TOR. Both policy processes faced challenges of limited government funding for policy development/revision activities. Under both policies, there were limited activities by independent external or internal teams to monitor and evaluate the policy development process and subsequently implementation/performance of the policy (Table 2). The program officers mentioned that beyond policy development and revision, policy implementation and performance were not formally monitored. ‘*After the launch of the policies by the MoH, there was no clear plan for the policy implementation*’ said one interviewee.

### Suggestions to improve the policy development/revision processes

The program officers made various recommendations to strengthen the policy development/revision systems at the ACP. The national guidelines were too broad and did not address activities in detail; for example: the processes of systematic review of evidence, criteria for selection of TWG, number of activities and timelines, monitoring and evaluation framework for each policy development/revision process. In addition, the technical officers recommended that the MoH policy analysis unit should play an active role especially in the pre-policy planning processes.

## Discussion

Overall, we found that similar processes were taken in the development and review of policies by the ACP and they were in accordance with the recommended procedure. A committee was set up and up to four drafts were developed before the actual launching of the policies. The authors felt that two years was too long a period for the revision of the HCT policy. The challenge of limited indigenous funding to support the planned activities in part contributed to the delayed timelines. On the other hand, one year for development of the SMC policy was not too off the mark although it was longer than the anticipated period of six months as per the TOR. In addition, the reported delays could be due to the lack of proper planning and documentation as implied by the absence of detailed protocols or standard operating procedures developed for each policy development/revision process to support the broad guideline document. Given that policy making largely includes government officials and local service managers [3], the government commitment should be reflected through committed resources [2,11] as well as timely utilization of the evidence-based policies; all of which are critical determinants of the quality of policy review processes and subsequently attainment of the desired health outcomes. Such government commitment provides a platform for strategic management of all stakeholders to facilitate contextualized and feasible policies. There is need for a robust evaluation system to understand the specific bottlenecks and help the policy unit to set appropriate timelines.

The issue of using TWG with individuals that have fulltime engagements elsewhere remains a challenge because these are often important stakeholders who should be part of the process. Yet, they may not have sufficient time for many meetings and to compile documents. This calls for good management to minimize the number of meetings and duration. Use of consultants to compile the suggestions from TWGs could ease the process and time demands on such members. On the other hand, through the policy analysis unit, the MoH could have fulltime staff to lead the specific policy development/evaluation/revision processes including systematic reviews [3,12], policy dialogues [4], policy briefs [7] and stakeholders meetings so that the TWG members get engaged to review and give expert opinions. This is clearly one of those issues that could be addressed by an independent team to monitor and evaluate the policy development, revision and implementation processes as a formal regulatory framework [11].

### Availability and utilization of national policy development and management guidelines

**Figure 1:**
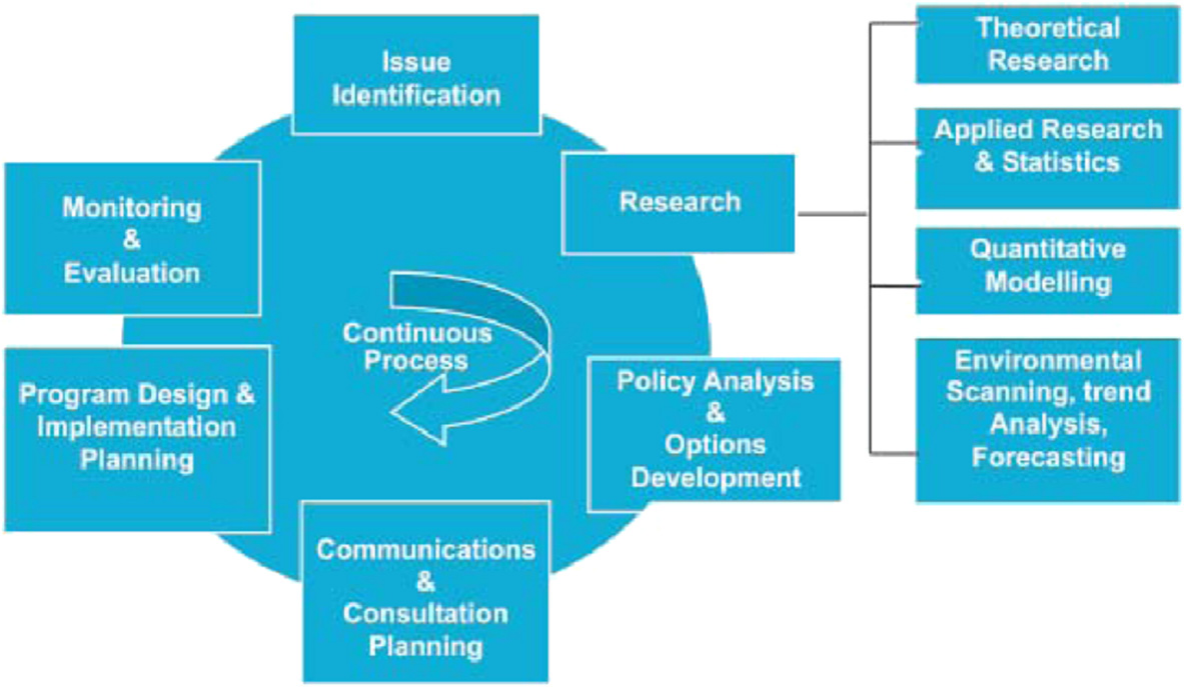
Policy development process adopted from the Uganda guide to policy development and management.

Given the limited awareness of the national policy development and management guidelines, we recommended a planned dissemination of the national policy guidelines to all the technical officers and their utilization in subsequent policy development and revision processes [10]. There is need to un-package the general policy development and management guidelines (Figure 1) into specific activities with timelines whenever the need arises to develop/revise a policy at the MoH. Therefore, we recommended active involvement of the policy analysis unit in the development of detailed policy development/revision protocols or SOPs (Table 3). In addition, there is need for a policy monitoring and evaluation plan to ensure consideration of key implementation elements such as prioritization, quality, costs, local acceptability, equity and consequences [4-8,13], identify bottlenecks and address the current challenges that cause unnecessary delays. This is likely to further improve the process and performance in the HIV response by the ACP.

**Table 3:** Proposed steps to strengthen the policy development/revision processes in AIDS control program, Uganda

**Planning processes in policy development/revision**	
**Policy analysis unit**	**Active involvement by the Ministry of Health (MOH) policy analysis unit**
**Policy development/revision**	**Policy development protocol and standard operating procedures to be developed and followed when need arises to develop/revise any policy**
	- Allocation of required resources by the MoH senior management team as per budget
	- List and set timelines for both minor and major activities with reference to the guidelines
	- Selection of technical working group (according to specific criteria) with clear terms of reference
	- Development of a policy implementation plan including an internal and external monitoring and evaluation plan
	- Develop a policy surveillance and impact assessment plan
**Policy approval**	**Prior approval of the policy development protocol by MoH senior management in consultation with the ACP technical team**

### Policy implementation, monitoring and evaluation

Lack of a formal regular post-policy evaluation and impact assessment plan is likely to affect the desired health outcomes of otherwise good policies. Some important positive and negative outcomes likely go undocumented thereby hindering optimal benefits from the policies [14]. Hence, the need to prioritize resources towards monitoring and evaluation of policy implementation processes as well as the actual impact of the policies both in the short- and long-term. We recommend establishment of independent internal or external monitoring and impact assessment processes to ensure dynamic and strategic implementation of evidence-based policies, as well as the impact on relevant health outcomes (Table 3).

### Implications of the study

This policy review exercise was locally driven and supported by the ACP program officers. In addition, we used appropriate methods such as key informant interviews [3]. Therefore, our recommendations are likely to be supported and implemented to improve the policy development and revision processes at the MoH. These results were disseminated to the ACP where we created awareness of the gaps that need to be filled. Investing in a consistent and comprehensive policy development and review process is likely to increase documentation of the outcomes of the national HIV response. This could potentially be applied to policy processes in other programs within the MoH.

### Limitations

Although we evaluated the presence of policy documents and their utilization, the quality of documents was not evaluated. It is likely that less than optimal guidelines and lack of an independent monitoring and evaluation system would compromise the policy development/revision, quality, uptake and health outcomes of the specific policies. We did not determine how much the policy development/revision process consumed in monetary terms and we did not evaluate policy implementation processes outside the MoH. Both of these elements are required to inform the establishment of comprehensive and sustainable policy development/revision systems.

## Conclusions

Policy development and revision of the Uganda SMC and HCT policies respectively, followed similar processes. Gaps identified include lack of protocols/SOPs for the processes and limited indigenous funding to support adherence to anticipated timelines; in addition to the busy technical working group members and lack of formal policy monitoring and evaluation processes. We recommend active involvement of the policy analysis unit in all policy processes. Specific protocols/SOPs for development, analysis, revision, implementation, monitoring, evaluation and impact assessment processes should be developed prior to commencement of the activities.

## Abbreviations

ACP: AIDS control program;HCT: HIV counseling and testing;MoH: Ministry of Health;SMC: Safe male circumcision;SOPs: Standard operating procedures;TOR: Terms of reference;TWG: Technical working group

## Competing interests

The authors declare that they have no competing interests.

## Authors’ contributions

BT, DN, RW, AZ and NS contributed to the conceptualization of the project. BT, RW and AZ contributed to the data collection. BT and DN contributed to the data analysis, interpretation and drafted the manuscript. All authors reviewed and approved the final draft of the manuscript for submission.
